# Mechanism of parkinsonian neuronal oscillations in the primate basal ganglia: some considerations based on our recent work

**DOI:** 10.3389/fnsys.2014.00074

**Published:** 2014-05-23

**Authors:** Atsushi Nambu, Yoshihisa Tachibana

**Affiliations:** ^1^Division of System Neurophysiology, National Institute for Physiological SciencesOkazaki, Japan; ^2^Department of Physiological Sciences, Graduate University for Advanced StudiesOkazaki, Japan

**Keywords:** Parkinson’s disease, neuronal oscillation, globus pallidus, subthalamic nucleus, β-band, monkey, basal ganglia

## Abstract

Accumulating evidence suggests that abnormal neuronal oscillations in the basal ganglia (BG) contribute to the manifestation of parkinsonian symptoms. In this article, we would like to summarize our recent work on the mechanism underlying abnormal oscillations in the parkinsonian state and discuss its significance in pathophysiology of Parkinson’s disease. We recorded neuronal activity in the BG of parkinsonian monkeys treated with 1-methyl-4-phenyl-1,2,3,6-tetrahydropyridine. Systemic administration of L-DOPA alleviated parkinsonian motor signs and decreased abnormal neuronal oscillations (8–15 Hz) in the internal (GPi) and external (GPe) segments of the globus pallidus and the subthalamic nucleus (STN). Inactivation of the STN by muscimol (GABA_A_ receptor agonist) injection also ameliorated parkinsonian signs and suppressed GPi oscillations. The blockade of glutamatergic inputs to the STN by local microinjection of a mixture of 3-(2-carboxypiperazin-4-yl)-propyl-1-phosphonic acid (glutamatergic NMDA receptor antagonist) and 1,2,3,4-tetrahydro-6-nitro-2,3-dioxo-benzo[f]quinoxaline-7-sulfonamide (glutamatergic AMPA/kainate receptor antagonist) suppressed neuronal oscillations in the STN. STN oscillations were also attenuated by the blockade of GABAergic neurotransmission from the GPe to the STN by muscimol inactivation of the GPe. These results suggest that cortical glutamatergic inputs to the STN and reciprocal GPe-STN interconnections are both important for the generation and amplification of the oscillatory activity of GPe and STN neurons in the parkinsonian state. The oscillatory activity in the STN is subsequently transmitted to the GPi and may contribute to manifestation of parkinsonian symptoms.

## Introduction

Parkinson’s disease (PD) is a neurodegenerative disorder affecting motor and non-motor functions. Motor dysfunction in PD, including akinesia, tremor and rigidity is largely attributed to the progressive loss of dopaminergic (DAergic) neurons in the substantia nigra pars compacta. There are two hypotheses that explain the pathophysiology of PD. The “firing rate model” originally proposed that dopamine (DA) depletion reduces tonic excitation to striatal neurons projecting to the internal segment of the globus pallidus (GPi) (i.e., *direct* pathway) and tonic inhibition to striatal neurons projecting to the external segment of the globus pallidus (GPe) (*indirect* pathway) (DeLong, 1990; Mallet et al., 2006). Both of these changes are thought to increase average firing rates of GPi and substantia nigra pars reticulata neurons. This increased activity in the basal ganglia (BG) output nuclei induces decreased activity in thalamic and cortical neurons, resulting in akinesia. However, recent electrophysiological studies using 1-methyl-4-phenyl-1,2,3,6-tetrahydropyridine (MPTP)-induced PD monkeys have failed to detect an expected increase in GPi activity (Wichmann et al., 1999; Raz et al., 2000; Rivlin-Etzion et al., 2008).

The firing rate model has now been largely supplanted by the “firing pattern model” that emphasizes oscillatory and/or synchronized activity. Oscillatory and/or synchronized activity is frequently observed in the BG of patients with movement disorders and animal models, which may cause the disturbance of information processing in the BG (Bergman et al., 1998). Unit activity and local field potentials recorded from PD animals and patients have shown oscillatory and synchronized activity in the GPe, GPi and subthalamic nucleus (STN; Bergman et al., 1998; Levy et al., 2000; Raz et al., 2000; Brown et al., 2001; Brown, 2007). The frequency bands include the tremor (4–9 Hz) and β (10–30 Hz) bands. The β-band oscillation may be a primary cause of akinesia, since the treatment of akinesia with drugs effectively suppresses the β-band oscillation. Recent studies also reported β-band synchronized activity in STN neurons of PD patients (Moshel et al., 2013), and correlation between the high β-band activity and freezing gate in PD patients (Toledo et al., 2014). Deep brain stimulation (DBS), which has been widely accepted as an effective therapeutic option of PD, is suggested to improve motor symptoms by activation of efferent fibers (Hashimoto et al., 2003), changes of oscillatory activity (Vitek, 2008) and/or decoupling STN-GPi oscillations (Moran et al., 2012). By contrast, in the course of MPTP-treatment of monkeys, the appearance of PD motor symptoms preceded that of oscillatory activity (Leblois et al., 2007), seeming to contradict the firing pattern model.

In this article, we would like to summarize our recent work on the mechanism regulating the abnormal BG oscillations (Tachibana et al., 2011) and discuss its significance in PD pathophysiology.

## Oscillatory activity in the BG of PD

**Figure 1:**
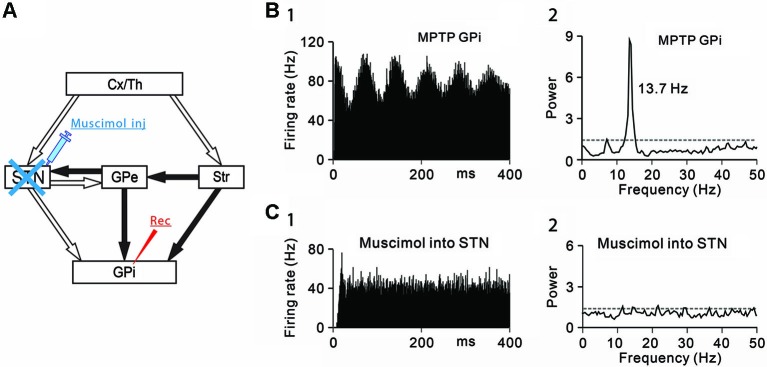
**Effects of subthalamic nucleus (STN) inactivation on neuronal activity of the internal segment of the globus pallidus (GPi) under the parkinsonian state. (A)** A schematic diagram showing anatomical connections of the basal ganglia and the experimental method. Recording from GPi neurons was performed with muscimol injection into the STN to block STN inputs to the GPi. Open and filled arrows represent glutamatergic and GABAergic projections, respectively. Cx, cerebral cortex; GPe, external segment of the globus pallidus; Str, striatum; Th, thalamus. **(B)** A representative GPi neuron showing abnormal oscillations in the parkinsonian state. **(1)** Autocorrelograms calculated from a 50-s spike train and **(2)** power spectra of the same spike trains are shown. Gray dashed lines represent a confidence level of *P* = 0.01. **(C)** Muscimol inactivation of the STN decreased the firing rate and 8–15 Hz oscillatory activity of the GPi neuron. Modified from Tachibana et al. (2011).

The firing properties of BG neurons were compared between the normal and PD states of macaque monkeys. PD states were induced by MPTP treatment (2.4–2.5 mg/kg, carotid artery injection and additional intravenous injections). The average firing rates of GPe neurons were significantly decreased (normal, 65.2 ± 25.8 Hz; PD, 41.2 ± 22.5 Hz) and those of STN neurons were significantly increased (normal, 19.8 ± 9.7 Hz; PD, 27.6 ± 11.4 Hz) in the PD state, whereas the firing rate of GPi neurons were not changed (normal, 67.0 ± 24.3 Hz; PD, 63.1 ± 26.9 Hz). These data contradict the firing rate model. Burst strength (Levy et al., 2001a; Wichmann and Soares, 2006) was increased in the GPi/GPe and STN of the PD states. The mean power (Soares et al., 2004; Rivlin-Etzion et al., 2006) of the 8–15 Hz (low-β) oscillations was increased in the GPi/GPe and STN, whereas there were no consistent changes in the 3–8 Hz and 15–30 Hz (high-β) oscillations. Oscillatory bursts of GPi/GPe and STN neurons were observed as multiple peaks in the autocorrelograms (e.g., Figures 1B1, 2B1, 3B1). The peak frequency with a maximum power of the oscillatory bursts of GPi/GPe and STN neurons was around 14 Hz (Figures 1B2, 2B2, 3B2).

**Figure 2:**
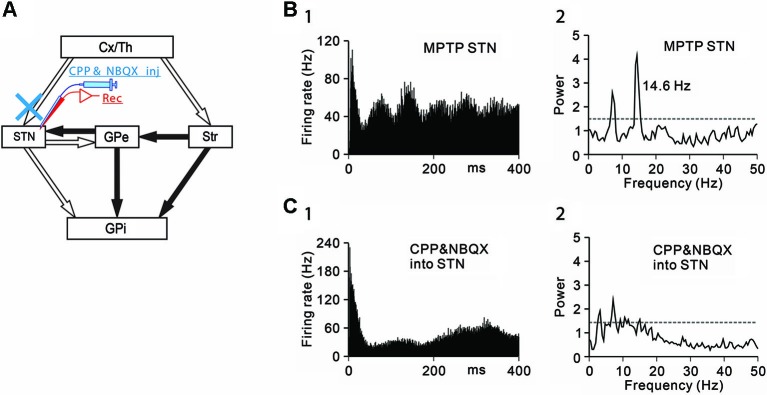
**Effects of the blockade of ionotropic glutamatergic inputs to STN neurons under the parkinsonian state. (A)** Recording from STN neurons was performed with intrasubthalamic microinjection of CPP and NBQX to block glutamatergic inputs to the STN. **(B)** A representative STN neuron showing abnormal oscillatory activity under the parkinsonian state. **(C)** Intrasubthalamic microinjection of CPP and NBQX decreased 3–8 Hz and 8–15 Hz oscillations of the STN neuron. Modified from Tachibana et al. (2011).

**Figure 3:**
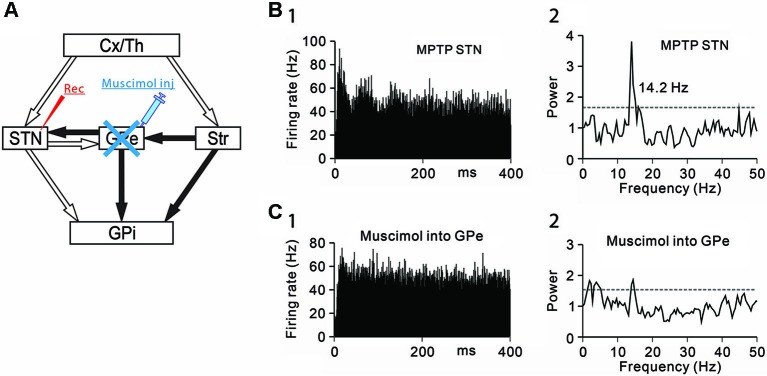
**Effects of GPe inactivation on STN neurons under the parkinsonian state**. **(A)** Recording from STN neurons was performed with muscimol injection into the GPe to block GABAergic inputs from the GPe. **(B)** A representative STN neuron showing abnormal 8–15 Hz oscillations under the parkinsonian state. **(C)** Muscimol inactivation of the GPe decreased the 8–15 Hz oscillations and increased the firing rate of the STN neuron. Modified from Tachibana et al. (2011).

## DA dependence of BG oscillations

We first tested whether the abnormal BG oscillations depend on DAergic inputs. DA was administrated systemically to PD monkeys, and the effects on the neuronal activity of GPi/GPe and STN neurons were examined. The motor disability was ameliorated within 5 min after intravenous L-DOPA injections (2.5–3.5 mg/kg, iv). L-DOPA administration decreased 8–15 Hz oscillations in the GPi/GPe and STN. Approximately 30 min after L-DOPA injections, the monkeys returned to the PD states, and the abnormal oscillations reappeared. The overall firing rate was not changed throughout the injections. These results have demonstrated that abnormal burst firing and 8–15 Hz oscillatory activity of GPi/GPe and STN neurons are DA-dependent. They also suggest that neuronal oscillations in the GPi/GPe and STN, rather than their spontaneous firing rate changes, may be critical for PD symptoms, supporting the firing pattern model.

## Origins of abnormal GPi/GPe oscillations

Then, the origins of 8–15 Hz GPi/GPe oscillations were examined. The GPi (Tachibana et al., 2008) and GPe (Kita et al., 2004) receive glutamatergic inputs from the STN and GABAergic inputs from the striatum and GPe (GPe-GPe projections via the intranuclear axon collaterals). To determine which inputs contribute to abnormal 8–15 Hz GPi oscillations, each input was selectively blocked. Firstly, the STN was inactivated by injection of a GABA_A_ receptor agonist, muscimol (4.4 mM, 0.5–1.0 µL) while GPi neuronal activity was simultaneously recorded (Figure 1A). Inactivation of the STN ameliorated PD motor signs, such as bradykinesia and rigidity, as previously reported (Bergman et al., 1990; Wichmann et al., 1994; Levy et al., 2001b) and decreased the 8–15 Hz oscillations (Figures 1B, C) and the firing rate.

Secondly, GABAergic inputs from the striatum and GPe were blocked, and the effects on the oscillatory activity of GPi/GPe neurons were examined. Microinjection of a GABA_A_ receptor antagonist, gabazine (1 mM, 0.1–0.2 µL) in the vicinity of recorded GPi/GPe neurons increased the firing rate of GPi/GPe neurons, and augmented the 8–15 Hz GPi oscillations, but induced no changes in GPe oscillations. These results suggest that 8–15 Hz GPi/GPe oscillations are generated by glutamatergic inputs mainly from the STN, but not by GABAergic inputs from the striatum and GPe.

## Origins of abnormal STN oscillations

Next, the origins of 8–15 Hz STN oscillations were examined. The STN receives glutamatergic inputs from the cerebral cortex and the thalamus, and GABAergic inputs from the GPe. Firstly, ionotropic glutamatergic inputs were blocked, and the effects on the oscillatory activity of STN neurons were examined (Figure 2A). Microinjection (0.1–0.2 µL) of a mixture of an *N*-methyl-D-aspartate receptor antagonist, 3-(2-carboxypiperazin-4-yl)-propyl-1-phosphonic acid (CPP, 1 mM) and an AMPA/kainate receptor antagonist, 1,2,3,4-tetrahydro-6-nitro-2,3-dioxo-benzo[f]quinoxaline-7-sulfonamide (NBQX, 1 mM) in the vicinity of recorded STN neurons decreased the 8–15 Hz oscillations (Figures 2B, C).

Secondly, GABAergic inputs from the GPe were blocked, and the effects on the oscillatory activity of STN neurons were examined (Figure 3A). Muscimol inactivation (1–2 µL) of the GPe attenuated the 8–15 Hz STN oscillations (Figures 3B, C) and increased the firing rate. However, the GPe inactivation induced no clear behavioral changes. These findings have shown that the 8–15 Hz STN oscillation are generated by glutamatergic inputs from the cortex and thalamus and GABAergic inputs from the GPe.

Previous studies reported the coherence between the electrocorticogram and the STN LFPs/STN unit activity in the PD state and have suggested that cortical glutamatergic inputs can drive STN oscillations in frequency bands below 30 Hz (Magill et al., 2000, 2001; Sharott et al., 2005; Mallet et al., 2008b). It is hypothesized that cortical β-rhythm is preferentially transmitted to the BG (Brittain and Brown, 2014). This idea is also supported by an optogenetic study that selective stimulation of cortico-STN projections ameliorated PD symptoms (Gradinaru et al., 2009). The other glutamatergic inputs to the primate STN may come from the intralaminar thalamic nuclei (Lanciego et al., 2009). The parafascicular thalamic nucleus (PF) neurons in PD rats showed oscillatory activity (0.5–2.5 Hz), but PF firings lagged STN firings (Parr-Brownlie et al., 2009).

Another origin of STN oscillations may be the GABAergic inputs from the GPe (Baufreton et al., 2005a). An *in vivo* rat study indicated that 15–30 Hz oscillations between GPe and STN neurons were developed during DA depletion (Mallet et al., 2008a). DAergic innervation in the GPe was decreased in PD monkeys (Schneider and Dacko, 1991), and the GPe-GPe GABAergic transmission was augmented (Watanabe et al., 2009). The oscillatory glutamatergic inputs mainly from the cortex and synchronized GABAergic inputs from the GPe may accelerate the oscillatory activity in STN neurons (Shen and Johnson, 2000, 2005; Baufreton et al., 2005b; Baufreton and Bevan, 2008).

## BG oscillations and PD pathophysiology

**Figure 4:**
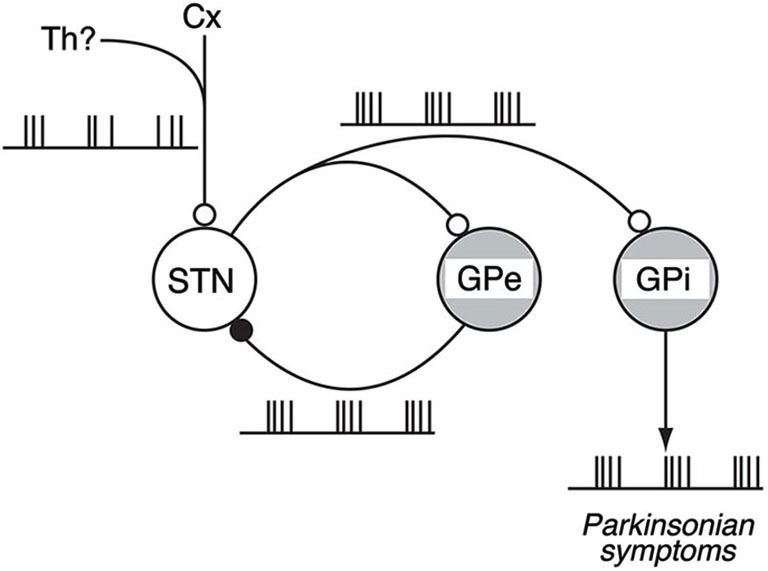
**Schematic diagram showing neural circuits involved in the generation of BG oscillations**. Under the parkinsonian sate, glutamatergic inputs from the Cx (and also from the Th) to the STN and reciprocal GPe-STN interconnections can cooperatively generate and amplify the oscillatory activity of STN and GPe neurons. Such oscillatory activity is subsequently transmitted to the GPi, contributing to the expression of parkinsonian symptoms. Open and filled circles represent glutamatergic and GABAergic synapses, respectively. Modified from Tachibana et al. (2011).

Our work shows the following results: (1) The loss of DA induced abnormal 8–15 Hz oscillations in GPi/GPe and STN neurons; (2) The abnormal 8–15 Hz GPi/GPe and STN oscillations were reversed by systemic DA administration; (3) The abnormal 8–15 Hz GPi/GPe oscillations were originated from the STN oscillations; and (4) The STN oscillations were driven by glutamatergic inputs mainly from the cortex and GABAergic inputs from the GPe. These findings support the firing pattern model and suggest the mechanism of BG oscillations: Glutamatergic inputs to the STN and reciprocal GPe-STN interconnections generate and amplify the oscillatory activity of STN and GPe neurons in PD. Such oscillatory activity is subsequently transmitted to GPi neurons, and finally reaches the thalamus, cortex and brain stem, contributing to the expression of PD symptoms (Figure 4).

The causal relationship between the BG oscillations and PD symptoms is a fundamental question. Leblois et al. (2007) have reported that oscillatory activity of BG neurons does not precede the appearance of PD motor symptoms in the course of chronic MPTP treatment of monkeys, questioning such causal relationship. Moreover, acute disruption of DA transmission did not develop oscillatory activity, which is distinct from chronically depleted animals (Mallet et al., 2008b). The BG oscillations may merely reflect other fundamental activity changes. In PD, the balance between the cortico-STN-GPi *hyperdirect* (Nambu et al., 2000), cortico-striato-GPi *direct* and cortico-striato-GPe *indirect* pathways was lost by the lack of DA in the striatum, and the “dynamic” network properties of the BG were changed (Nambu et al., 2005; Kita and Kita, 2011). It is suggested that the imbalance between the *hyperdirect* and *direct* pathways generates the BG oscillations (Leblois et al., 2006). Further studies are needed to solve this fundamental question.

In this article, we would like to emphasize a close relationship between the BG oscillations and PD symptoms. In fact, DAergic medication, STN-DBS, and voluntary movements in human patients are all reported to decrease the cortico-BG synchronization (Brown et al., 2001, 2004; Cassidy et al., 2002; Levy et al., 2002; Williams et al., 2002; Silberstein et al., 2005; Lafreniere-Roula et al., 2010). In a similar manner, the suppression of 8–15 Hz oscillations in the primate BG may be essential to ameliorate PD motor symptoms. These findings could shed light on the pathophysiology of PD and understanding the mechanisms of current therapies, and lead us to further rational treatments of PD.

## Conflict of interest statement

The authors declare that the research was conducted in the absence of any commercial or financial relationships that could be construed as a potential conflict of interest.
